# Immune responses to oral poliovirus vaccine in HIV-exposed uninfected Zimbabwean infants

**DOI:** 10.1080/21645515.2017.1359454

**Published:** 2017-08-31

**Authors:** James A. Church, Sandra Rukobo, Margaret Govha, Marya P. Carmolli, Sean A. Diehl, Bernard Chasekwa, Robert Ntozini, Kuda Mutasa, Jean H. Humphrey, Beth D. Kirkpatrick, Andrew J. Prendergast

**Affiliations:** a Zvitambo Institute for Maternal and Child Health Research, Harare, Zimbabwe; b Blizard Institute, Queen Mary University of London, London, UK; c Department of Medicine, Vaccine Testing Center, University of Vermont, Burlington, VT, USA; d Department of International Health, Johns Hopkins Bloomberg School of Public Health, Baltimore, MD, USA

**Keywords:** Africa, HIV, infants, poliovirus, oral vaccine, OPV

## Abstract

It remains uncertain whether HIV-exposed uninfected (HEU) infants have impaired responses to oral vaccines. We performed a cross-sectional study of 6-month-old infants recruited at birth to the ZVITAMBO trial in Zimbabwe between 1997–2001, before introduction of prevention of mother-to-child transmission interventions. We measured poliovirus-specific IgA to type 1–3 polio strains by semi-quantitative capture ELISA in cryopreserved serum samples collected from 85 HEU and 101 HIV-unexposed infants at 6 months of age, one month after their last immunisation with trivalent OPV. Almost all infants were breastfed, with the majority in both groups mixed breastfed (70.6% HEU versus 71.3% HIV-unexposed). Median (IQR) vaccine titers for HEU and HIV-unexposed infants were 1592 (618–4896) vs. 1774 (711–5431) for Sabin 1 (*P* = 0.46); 1895 (810–4398) vs. 2308 (1081–4283) for Sabin 2 (*P* = 0.52); and 1798 (774–4192) vs. 2260 (996–5723) for Sabin 3 (*P* = 0.18). There were no significant differences in vaccine titers between HEU and HIV-unexposed infants, suggesting that vertical HIV exposure does not impact oral poliovirus vaccine immunogenicity.

## Introduction

As coverage of prevention of mother-to-child transmission (PMTCT) increases, the number of HIV-infected infants is declining and there is a growing population of infants in sub-Saharan Africa who are HIV-exposed but uninfected (HEU).^1^ HEU infants appear to have higher morbidity and mortality and worse growth than HIV-unexposed infants,^2^ with evidence of altered immunity including diminished antibody responses to specific antigens.^3^^,^^4^ It is therefore plausible that inadequate vaccine responses might contribute to the increased infectious morbidity and mortality reported in HEU infants, although the evidence to date is limited and heterogeneous.^5^

Oral poliovirus vaccine (OPV) is a live attenuated vaccine, which is inexpensive and easy to administer. By acting in the gastrointestinal tract, OPV can interrupt transmission of the virus and therefore remains a key component of the Polio Eradication and Endgame Strategic Plan.^6^ However, seroconversion to OPV is lower in developing compared with developed countries,^7^ which has contributed to the challenges in fully eradicating polio.^8^ The reasons for this gap in performance are uncertain but may include environmental enteric dysfunction, malnutrition, interference from breast milk antibodies, co-administration with other oral vaccines and concurrent infections.^9-13^

HIV infection has been associated with significantly lower seroconversion rates to OPV^14^; however, it is unclear whether HIV exposure contributes to poor oral vaccine immunogenicity. The purpose of this study was therefore to evaluate immune responses to OPV in a well-characterized cohort of HEU and HIV-unexposed infants in Zimbabwe.

## Results

A total of 85 HEU and 101 HIV-unexposed infants fulfilled inclusion criteria. Baseline characteristics of infants and their mothers are shown in Table 1. Mothers of HEU infants were older than mothers of HIV-unexposed infants (26.4 vs. 24.6 years, respectively; *P* = 0.02) and had higher parity (3 vs. 2; *P* = 0.01); there were no other significant differences between groups. Almost all infants were breastfed, with the majority in both groups mixed breastfed (70.6% HEU vs. 71.3% HIV-unexposed); exclusive breastfeeding was low overall (7.1% vs. 5.0%, respectively).

**Table 1.** Baseline characteristics of infants and their mothers.

	HIV exposed uninfected (HEU) N = 85	HIV-unexposed N = 101	P value
**Infant Characteristics**			
Male sex, % (n)	56.5 (48)	52.5 (53)	0.66
Gestational age, weeks; mean (SD)	39.4 (1.6)	39.2 (2.1)	0.48
Birth weight, kg; mean (SD)	2.99 (0.47)	3.01 (0.44)	0.77
Birth length, cm; mean (SD)	48.4 (2.7)	47.8 (2.4)	0.11
Birth head circumference, cm; mean (SD)	34.2 (2.2)	34.1 (2.1)	0.75
Normal vaginal delivery, % (n)	87.0 (74)	86.7 (85) [98]	0.68
Exclusive breast feeding^1^, % (n)	7.1 (6)	5.0 (5)	0.55
Predominant breast feeding, % (n)	22.4 (19)	23.8 (24)	0.86
Mixed feeding, % (n)	70.6 (60)	71.3 (72)	1.00
**Maternal characteristics**			
Age, years; mean (SD)	26.4 (4.9)	24.6 (5.7)	0.02
Married or stable union, % (n)	91.8 (78)	95.0 (96)	0.39
Education, years; median (IQR)	10 (7,11)	11 (9,11)	0.14
Parity, median (IQR)	3 (2,3)	2 (1,3)	0.01
Maternal MUAC, cm; mean (SD)	26.4 (2.8)	26.4 (3.1)	1.00
Employed, % (n)	77.6 (66)	86.1 (87)	0.18
Monthly household income, USD; mean (SD)	7.83 (6.24)	7.31 (5.56)	0.56

[x] refers to total number if data missing

1 Breastfeeding status assessed at 6-months postpartum

**Figure 1.**
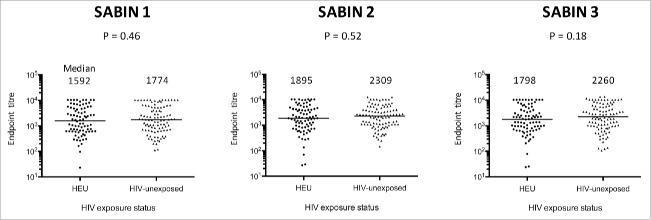
Serum polio-specific IgA end point titers (Sabin 1,2 and 3) at 6 months of age in HIV-exposed uninfected (HEU) and HIV-unexposed infants.

At 6 months of age (1 month post-immunisation), median (IQR) vaccine titers for HEU and HIV-unexposed infants were 1592 (618–4896) vs. 1774 (711–5431) for Sabin 1 (*P* = 0.46); 1895 (810–4398) vs. 2308 (1081–4283) for Sabin 2 (*P* = 0.52); and 1798 (774–4192) vs. 2260 (996–5723) for Sabin 3 (*P* = 0.18) (Fig. 1). Differences in log mean titers between HEU and HIV-unexposed groups were similar after adjusting for breastfeeding, birth weight and sex in a linear regression model (*P* = 0.36, Sabin 1; *P* = 0.33, Sabin 2; *P* = 0.19, Sabin 3).

## Discussion

Although immunological abnormalities have been described in HIV-exposed uninfected infants,^5^ our results suggest that immune responses to OPV were similar among HEU and HIV-unexposed Zimbabwean infants at 6 months of age, adding to the growing body of literature on vaccine responses in HEU infants.^1^

Most prior studies have evaluated responses to parenteral vaccines, generally finding similar (or even better) responses among HEU compared with HIV-unexposed infants.^15-18^ Few studies to date have evaluated responses to oral vaccines, and only 3 to our knowledge have compared OPV responses.^19-21^ A study of HEU infants in Zambia showed lower mean OPV 2 neutralising antibody titers at 18 months of age compared with HIV-unexposed infants, although this was not significant after adjusting for differences in breastfeeding.^20^ HEU infants in Cameroon and Central African Republic aged 18–35 months showed high rates of seroconversion to trivalent OPV (96–100%).^21^ A study from Malawi found no difference in median anti-polio immunoglobulin G (IgG) titers between HEU and HIV-unexposed infants at 10 weeks of age, but only included 34 infants.^19^ Our study of HEU infants from Zimbabwe is consistent with these prior studies, showing that OPV was equally immunogenic in HEU and HIV-unexposed infants.

A major strength of this study is the well-characterized cohort of HEU infants born in the pre-PMTCT era together with a demographically similar HIV-unexposed population. However, there are a few important limitations. Firstly, immunisation data were not collected as part of the original ZVITAMBO trial, so we were unable to confirm the number of OPV doses received by infants. However, we have no reason to believe that HEU infants would be less likely to complete the EPI schedule than HIV-unexposed infants, and in fact clinic attendance for intercurrent illnesses was higher among HEU infants.^22^ Secondly, our sample size was limited by the number of cryopreserved specimens with sufficient volume still remaining from the original trial. We may have therefore been underpowered to pick up differences between groups. Thirdly, while the ELISA we used to detect polio-specific IgA enables a comparison of immunogenicity between groups, we did not have access to the gold-standard OPV neutralization assay, which provides a measure of vaccine protection. Fourth, without available samples to measure baseline IgA titers, we cannot rule out a difference between groups in antibody rise between pre- and post-vaccination. Finally, we only measured antibody responses shortly after immunisation. There is some evidence that the quality and quantity of vaccine-specific antibody declines faster in HEU compared with HIV-unexposed infants^23^ so we cannot comment on durability of protection.

In summary, in a well-characterized cohort of HEU and HIV-unexposed infants, recruited before PMTCT introduction, we found that HIV exposure does not appear to affect serum IgA responses to OPV. In an era when new oral vaccines, such as rotavirus, are being introduced in areas of high antenatal HIV prevalence, this is reassuring, although further studies are required to ascertain the reasons for the recognized underperformance of oral vaccines in developing countries.

## Materials and methods

### Study design and population

This study utilised archived samples from the Zimbabwe Vitamin A for Mothers and Babies (ZVITAMBO) trial, which has been described previously.^24^ Briefly, 14110 mother-infant pairs were enrolled within 96 hours of delivery in Harare, Zimbabwe between 1997 and 2001. Mother-infant pairs were eligible if neither had an acutely life-threatening condition and the infant was a singleton with birth weight >1500 g. Written informed consent was obtained. Socioeconomic and demographic information was collected by maternal interview. Follow-up was conducted at 6 weeks, 3 months and then 3 monthly to 12–24 months of age. The trial was conducted in a peri-urban setting and preceded availability of antiretroviral therapy in Zimbabwe or use of cotrimoxazole prophylaxis for HIV-exposed infants. Anthropometry was conducted at each visit, using methods and WHO reference standards as described previously.^25^

### Biological specimen collection

Blood was collected by venipuncture from all mothers and infants at baseline (≤96 hours after delivery) and at all follow-up visits. Samples were centrifuged and plasma removed within 2 hours of collection. Samples were stored in −80°C freezers with automatic generator backup

### Ascertainment of HIV exposure status

Mothers underwent HIV testing at baseline using 2 parallel ELISA assays. Women testing HIV-negative were re-tested at every visit to detect HIV seroconversion. The last available sample from each child was tested for HIV by GeneScreen ELISA on plasma if aged ≥ 18 months, or by DNA polymerase chain reaction (Roche Amplicor version 1.5; Roche Diagnostic Systems, Alameda, CA) in cell pellets if aged <18 months. If the last available sample was negative, the child was classified as HIV-negative. Children were classified as HIV-unexposed if the mother tested HIV-negative at baseline and did not seroconvert during follow-up; children were classified as HIV-exposed uninfected (HEU) if the mother tested HIV-positive at baseline and the last available infant sample was HIV-negative. Infants of mothers who seroconverted during follow up were not included.

### Selection of infants

For the current cross-sectional study, we retrieved samples for all HEU and HIV-unexposed infants fulfilling the following criteria: 1) Mother and infant both received placebo in the original trial; 2) infants were followed to 6 months with available feeding and anthropometry data; and 3) a sufficient sample of cryopreserved serum was available at 6 months of age.

### Infant feeding counselling

All mothers, irrespective of HIV status, were encouraged to exclusively breastfeed their infants for 6 months. Data on feeding practices at 6 weeks and 3 months were used to categorise infants as exclusively, predominantly or mixed breastfed, as previously defined.^26^

### Oral poliovirus vaccination

Infants followed the routine Expanded Programme of Immunisation schedule in Zimbabwe at the time, which included trivalent OPV at 3, 4 and 5 months of age. Specific vaccination data were not collected as part of the trial, but OPV3 immunisation coverage (collated by WHO using data from routine reporting systems) at that time in Zimbabwe was 70–81%.^27^

### Antigen-capture ELISA for detection of poliovirus IgA

Although neutralising antibody titer is the best correlate of protection for OPV,^28^^,^^29^ measurements of mucosal (salivary/stool secretory IgA) and circulating (serum) IgA responses to poliovirus are useful in the detection and control of poliovirus infection,^30-35^ particularly in settings without access to a WHO-accredited polio reference laboratory capable of performing neutralization assays.

In all 6-month serum samples (i.e. one month after receipt of final OPV), we measured poliovirus-specific IgA to type 1–3 polio strains in a capture ELISA developed by collaborators at the Centers for Disease Control and Prevention (CDC) as described previously.^36^ First, 96-well microplates were coated with goat anti-human IgA (SeraCare, Massachusetts) for 60 minutes at 37°C. Next plates were washed with buffer containing phosphate buffered saline (PBS) and 0.05% Tween 20 and incubated for a further 60 minutes with dilution buffer containing bovine serum albumin dissolved in PBS (all purchased from Sigma, St Louis). We then made 3-fold serial dilutions of serum samples beginning at a dilution of 1 in 100 and added 50 uL of diluted sample to each well (one patient sample per column). After a further wash step, the plates were incubated overnight in a moist chamber at room temperature with Sabin antigen, cultured from Hep2C cells at the University of Vermont. The next day, the plates were washed followed by addition of monoclonal Sabin antibody (Merck Millipore, Massachusetts) to the corresponding Sabin antigen and incubated at 37°C for 60 minutes. After another wash step, goat anti-mouse IgG conjugated to enzyme (SeraCare) was added to the plates for a further 60 minutes at 37°C. We then developed the reactions by addition of substrate for 15 minutes at room temperature, with the output being optical densitiy (O.D.) at 450 nm. Pooled serum from poliovirus-vaccinated healthy donor volunteers was used as a positive control; each plate also included a blank column with no test sample as a negative control.

Using 3-fold serial dilutions of patient samples run out in a single column allowed for a semi-quantitative measure of poliovirus-specific IgA, an adaptation of the original method. Endpoint titers were calculated by subtracting the plate background (i.e., the average O.D. of the negative control wells), then identifying the dilution of the final well in each sample column with an O.D. >0.07 (a 95% confidence value cut-off used to distinguish “negative” from “positive” absorbance values and calculated according to the original method^36^). The intra- and inter-plate coefficients of variation for Sabin 1, 2 and 3 strains, derived from the mean O.D.s and standard deviations from 7 successive preliminary experiments run before including study samples, were 8.2%, 4.0% and 4.8% and 20.1%, 17.7% and 16.8%, respectively. As a means of continued quality assurance, the end point titer of the positive control sample in all subsequent assays had to fall within an acceptable range for the experiment to be deemed valid. The range was pre-determined based on the upper and lower limit O.D.s in the 7 preliminary experiments. Laboratory scientists were blinded to infant HIV exposure status when conducting the assays.

If an end point titer could not be derived at the first attempt (e.g. bottom well O.D. ≥0.08), the assay was repeated using a 10-fold higher or lower concentration of patient sample where sufficient serum volume was available. If an end point titer could not be derived using the new sample concentration, the lowest or highest dilution factor was taken as the final end point. Extreme low and high end point titers were subsequently truncated and assigned a value equivalent to the 5th and 95th centile within the data set respectively.

### Statistical analysis

Baseline characteristics were compared between groups using Fisher's exact tests for categorical variables, and Mann-Whitney or 2-sample t-tests for continuous variables, depending on data distribution. A regression model was used to calculate adjusted differences between median vaccine titers between groups, using breastfeeding, birth weight and infant sex as covariates. Statistical analyses were performed using STATA 13 (Stata-Corp, College Station, TX, USA) and Prism version 6 (GraphPad Software Inc., La Jolla, CA, USA).

### Ethics statement

The original ZVITAMBO trial and this laboratory substudy were approved by the Medical Research Council of Zimbabwe, John Hopkins School of Public Health Committee on Human Research and the Montreal General Hospital Ethics Committee.
